# Alcohol drinking and head and neck cancer risk: the joint effect of intensity and duration

**DOI:** 10.1038/s41416-020-01031-z

**Published:** 2020-08-24

**Authors:** Gioia Di Credico, Jerry Polesel, Luigino Dal Maso, Francesco Pauli, Nicola Torelli, Daniele Luce, Loredana Radoï, Keitaro Matsuo, Diego Serraino, Paul Brennan, Ivana Holcatova, Wolfgang Ahrens, Pagona Lagiou, Cristina Canova, Lorenzo Richiardi, Claire M. Healy, Kristina Kjaerheim, David I. Conway, Gary J. Macfarlane, Peter Thomson, Antonio Agudo, Ariana Znaor, Silvia Franceschi, Rolando Herrero, Tatiana N. Toporcov, Raquel A. Moyses, Joshua Muscat, Eva Negri, Marta Vilensky, Leticia Fernandez, Maria Paula Curado, Ana Menezes, Alexander W. Daudt, Rosalina Koifman, Victor Wunsch-Filho, Andrew F. Olshan, Jose P. Zevallos, Erich M. Sturgis, Guojun Li, Fabio Levi, Zuo-Feng Zhang, Hal Morgenstern, Elaine Smith, Philip Lazarus, Carlo La Vecchia, Werner Garavello, Chu Chen, Stephen M. Schwartz, Tongzhang Zheng, Thomas L. Vaughan, Karl Kelsey, Michael McClean, Simone Benhamou, Richard B. Hayes, Mark P. Purdue, Maura Gillison, Stimson Schantz, Guo-Pei Yu, Shu-Chun Chuang, Paolo Boffetta, Mia Hashibe, Amy Lee Yuan-Chin, Valeria Edefonti

**Affiliations:** 1 Department of Economics, Business, Mathematics and Statistics, University of Trieste, Trieste, Italy; 2 Cancer Epidemiology Unit, Centro di Riferimento Oncologico di Aviano (CRO) IRCCS, Aviano, Italy; 3 Université de Rennes, INSERM, EHESP, Irset (Institut de Recherche en Santé, Environnement et Travail), UMR_S 1085, Pointe-à-Pitre, France; 4 INSERM UMR 1018, Centre for Research in Epidemiology and Population Health (CESP), Cancer Epidemiology, Genes and Environment Team, Villejuif, France; 5 Division of Cancer Epidemiology and Prevention, Aichi Cancer Center Research Institute, Nagoya, Japan; 6 Department of Cancer Epidemiology, Nagoya University Graduate School of Medicine, Nagoya, Japan; 7 International Agency for Research on Cancer, Lyon, France; 8 Institute of Hygiene & Epidemiology, 1st Faculty of Medicine, Charles University, Prague, Czech Republic; 9 Leibniz Institute for Prevention Research and Epidemiology, BIPS, Bremen, Germany; 10 University of Bremen, Faculty of Mathematics and Computer Science, Bremen, Germany; 11 Department of Hygiene, Epidemiology and Medical Statistics, School of Medicine, National and Kapodistrian University of Athens, Athens, Greece; 12 University of Padua, Padova, Italy; 13 Department of Medical Sciences, University of Turin, Turin, Italy; 14 Trinity College School of Dental Science, Dublin, Ireland; 15 Cancer Registry of Norway, Oslo, Norway; 16 School of Medicine, Dentistry, and Nursing, University of Glasgow, Glasgow, UK; 17 School of Medicine, Medical Sciences and Nutrition, University of Aberdeen, Aberdeen, UK; 18 University of Hong Kong, Hong Kong, China; 19 Unit of Nutrition and Cancer, Catalan Institute of Oncology - ICO, Nutrition and Cancer Group, Bellvitge Biomedical Research Institute—IDIBELL, L’Hospitalet de Llobregat, Barcelona, 08908 Spain; 20 Scientific Directorate, Centro di Riferimento Oncologico di Aviano (CRO) IRCCS, Aviano, Italy; 21 Department of Epidemiology, School of Public Health, University of São Paulo, São Paulo, Brazil; 22 Head and Neck Surgery, School of Medicine, University of São Paulo, São Paulo, Brazil; 23 Penn State College of Medicine, Hershey, PA USA; 24 Department of Biomedical and Clinical Sciences, University of Milan, Milan, Italy; 25 Instituto de Oncología Ángel H. Roffo, Universidad de Buenos Aires, Buenos Aires, Argentina; 26 Institute of Oncology and Radiobiology, Havana, Cuba; 27 Epidemiology—CIPE/ACCAMARGO, Sao Paulo, Brazil; 28 Universidade Federal de Pelotas, Pelotas, Brazil; 29 Hospital Moinhos de Vento, Porto Alegre, Brazil; 30 Escola Nacional de Saude Publica, Fundacao Oswaldo Cruz, Rio de Janeiro, Brazil; 31 Department of Epidemiology, Gillings School of Global Public Health, Chapel Hill, NC USA; 32 Division of Head and Neck Surgical Oncology in the Department of Otolaryngology/Head and Neck Surgery at Washington University School of Medicine, St Louis, MO USA; 33 Department of Otolaryngology-Head and Neck Surgery, Baylor College of Medicine, Houston, Texas USA; 34 Institut Universitaire de Médecine Sociale et Préventive (IUMSP), Unisanté, University of Lausanne, Lausanne, Switzerland; 35 UCLA School of Public Health, Los Angeles, CA USA; 36 Departments of Epidemiology and Environmental Health Sciences, School of Public Health and Department of Urology, Medical School, University of Michigan, Ann Arbor, MI USA; 37 College of Public Health, University of Iowa, Iowa City, IA USA; 38 Department of Pharmaceutical Sciences, College of Pharmacy and Pharmaceutical Sciences, Washington State University, Spokane, WA USA; 39 Branch of Medical Statistics, Biometry and Epidemiology “G. A. Maccacaro”, Department of Clinical Sciences and Community Health, Università degli Studi di Milano, Milano, Italy; 40 Department of Otorhinolaryngology, School of Medicine and Surgery, University of Milano—Bicocca, Monza, Italy; 41 Program in Epidemiology, Division of Public Health Sciences, Fred Hutchinson Cancer Research Center, Seattle, WA USA; 42 Department of Epidemiology, Brown University School of Public Health, Providence, RI USA; 43 Brown University, Providence, Rhode Island, RI USA; 44 Boston University School of Public Health, Boston, MA USA; 45 National Institute of Health and Medical Research, INSERM U1018, Villejuif, France; 46 Division of Epidemiology, New York University School Of Medicine, New York, NY USA; 47 Division of Cancer Epidemiology and Genetics, National Cancer Institute, Bethesda, MD USA; 48 “Thoracic/Head and Neck Medical Oncology”, The University of Texas MD Anderson Cancer Center, Houston, TX USA; 49 New York Eye and Ear Infirmary, New York, NY USA; 50 Medical Informatics Center, Peking University, Beijing, China; 51 Institute of Population Health Sciences, National Health Research Institutes, Miaoli, Taiwan; 52 Department of Medical and Surgical Sciences, University of Bologna, Bologna, Italy; 53 The Tisch Cancer Institute, Icahn School of Medicine at Mount Sinai, New York, NY USA; 54 Division of Public Health, Department of Family & Preventive Medicine, University of Utah School of Medicine and Huntsman Cancer Institute, Salt Lake City, UT USA

**Keywords:** Risk factors, Diseases

## Abstract

**Background:**

Alcohol is a well-established risk factor for head and neck cancer (HNC). This study aims to explore the effect of alcohol intensity and duration, as joint continuous exposures, on HNC risk.

**Methods:**

Data from 26 case-control studies in the INHANCE Consortium were used, including never and current drinkers who drunk ≤10 drinks/day for ≤54 years (24234 controls, 4085 oral cavity, 3359 oropharyngeal, 983 hypopharyngeal and 3340 laryngeal cancers). The dose-response relationship between the risk and the joint exposure to drinking intensity and duration was investigated through bivariate regression spline models, adjusting for potential confounders, including tobacco smoking.

**Results:**

For all subsites, cancer risk steeply increased with increasing drinks/day, with no appreciable threshold effect at lower intensities. For each intensity level, the risk of oral cavity, hypopharyngeal and laryngeal cancers did not vary according to years of drinking, suggesting no effect of duration. For oropharyngeal cancer, the risk increased with durations up to 28 years, flattening thereafter. The risk peaked at the higher levels of intensity and duration for all subsites (odds ratio = 7.95 for oral cavity, 12.86 for oropharynx, 24.96 for hypopharynx and 6.60 for larynx).

**Conclusions:**

Present results further encourage the reduction of alcohol intensity to mitigate HNC risk.

## Background

Worldwide, harmful alcohol consumption causes 3 million deaths each year (5% of all deaths), and it is responsible for ~5% of the global burden of disease and injury.^1^ In particular, alcohol consumption has been consistently associated with cancer risk at several sites.^2^ Together with tobacco smoking, alcohol drinking is one the major risk factors for head and neck cancer (HNC), and it is responsible for approximately one third of the cases worldwide.^3,4^

Epidemiological studies firmly established a clear dose-response relationship between ethanol intake and HNC risk.^5,6^ However, alcohol drinking has two related dimensions impacting on health outcomes: besides the quantity of alcohol consumed, time-related patterns of consumption, such as age at starting and duration, have a relevant role^1^ and they may modify the reported association between drinking intensity and cancer risk. Notably, a joint effect of intensity and duration on cancer risk has already been reported for tobacco smoking in HNC^7,8^ and in other tobacco-related cancers.^9–11^

In a previous analysis from the International Head and Neck Cancer Epidemiology (INHANCE) consortium including 15 studies,^7^ the independent contribution of drinking intensity and duration was estimated through the calculation of drink-years. Similarly to pack-years for tobacco smoking, drink-years represent the lifetime cumulative exposure to alcohol, and it was obtained by multiplying intensity (in drinks/day) by duration (in years). Then, the effect of duration on HNC risk was estimated analysing the risk for drink-years within fixed categories of intensity; this analysis reported an independent effect of drinking duration for all HNC subsites.^7^

In the absence of a clear relationship between alcohol intensity and duration on the risk of HNC, we investigated their joint effect on the INHANCE database using an extension of the bivariate spline model presented in a previously published analysis on cigarette smoking.^8^ Differently from the previous INHANCE paper,^7^ this model allows risks to vary for different combinations of drinking intensity and duration, even when the cumulative drink-years exposure is the same. We will address the following research questions: (1) What are the relationships between intensity and duration of alcohol drinking and the risk of cancer at HNC subsites? (2) Do drinking intensity and duration have a similar impact on HNC risk? (3) Are there meaningful values of drinking intensity or duration where the risk pattern changes?

## Methods

The INHANCE Consortium was established in 2004 to elucidate the aetiology of HNC through pooled analyses of individual-level data from several studies on a large scale.^12,13^ It included invasive cancer cases of the oral cavity, oropharynx, hypopharynx, oral cavity or pharynx not otherwise specified, larynx or unspecified HNC. Cases with cancers of the salivary glands or of the nasal cavity/ear/paranasal sinuses were excluded.^14^

At the time of this analysis, the INHANCE database (version 1.5) included 25,716 HNC cases and 37,111 controls (http://www.inhance.utah.edu, last access 25 May 2020). The present analysis was restricted to 26 case-control studies (21,384 HNC cases; 30,651 controls) that collected information on alcohol drinking status (i.e. never, former and current), intensity (number of drinks/day) and duration (years) at individual level (Supplementary Table S1).^15–40^ Cancer sites were grouped according to similar major aetiology: oral cavity (ICD10 codes: C02–C06; *n* = 6249), oropharynx (ICD10: C01, C09–C10; *n* = 5499), hypopharynx (ICD10: C13; *n* = 1798), and larynx (ICD10: C32; *n* = 5620). The following exclusion criteria were applied: (a) cancers arising in sites other than those mentioned above, or mixed cancer subsites (2218 subjects); (b) missing information on drinking status, intensity or duration (2247 subjects); (c) being former drinkers (i.e. having stopped drinking for at least 1 year before cancer diagnosis or interview for controls; 6993 subjects), as these subjects are more likely to stop drinking for reasons related to medical conditions;^41^ (d) missing information on major covariates, namely sex, age, education, ethnicity (92 subjects) or on cigarette-smoking status, intensity or duration (392 subjects) (see the flow-chart in Supplementary Fig. S1). In 15 studies^15–17,19,21,22,24,25,27,28,33,34,37,38,40^ controls were selected among cancer-free patients admitted to hospital for non-oncologic reasons, whereas controls were from the general population in nine studies;^18,20,23,29–32,36,39^ two multicentre studies^26,35^ enrolled a combination of hospital and population controls.

To prevent potential estimation distortion due to sparse data or misclassification at the highest levels of the exposure distributions, we further excluded subjects who reported the highest 5% of drinking intensity (i.e. >10 drinks/day) or duration (i.e. >54 years); consequently, 2455 HNC cases (17.3%) and 1637 controls (6.3%) were excluded. Finally, the current analysis included 4085 individuals with cancers of the oral cavity, 3359 oropharynx, 983 hypopharynx, 3340 larynx and 24,234 controls (Supplementary Table S2). For those studies reporting a case-control matching, separate sets of controls were matched for the three cancer subsites. Informed consent was obtained from all study subjects (Supplementary Table S1). The investigations were approved by the relevant Boards of Ethics, according to the regulation in force at time the data were collected.

Available data were harmonised at the Study Coordinating Center.^14^ While different studies had used different definitions of alcohol drinking status, the current paper defined as never drinkers those individuals who have never had any alcohol (0 ml of ethanol or 0 drinks over lifetime) or were defined as never drinkers by the individual studies. A similar definition was adopted for smoking habits.^8^ Study subjects were asked to report their drinking habits (drinking status, intensity and duration). Drinking intensity was then expressed in drinks/day of alcoholic beverages. To account for variation of ethanol content across alcoholic beverages and across countries, intensity was harmonised on a standard drink, corresponding to 15.6 ml (i.e. 12 g) of ethanol, weighting intensity by study-specific beverages volume and ethanol intake.^14^ Average lifetime alcohol intake was calculated as the total intake of wine, beer and hard liquor, taking into account possible intensity modification or quitting periods occurring in subjects’ life. Duration of alcohol drinking was calculated as the period of time between the subject’s age at the start of drinking any alcoholic beverages and the age at cancer diagnosis (or interview, for controls), discarding periods when the subject abstained from any alcoholic beverages.

The dose-response relationship between cancer risk and the joint exposure to alcohol drinking intensity and duration in current drinkers was investigated through bivariate regression spline models,^42^ as described elsewhere.^8,43^ In contrast to drink-years, this method allows risks to vary for different combinations of the two continuous exposures intensity and duration, even when the cumulative drink-year exposure is the same (i.e. people drinking 1 drink/day for 10 years are allowed to have a different risk than those drinking 10 drinks/day for one year). Briefly, within a generalised semi-parametric logistic regression model, the two exposures were entered as a joined piecewise polynomial of a linear degree with constraints for continuity at each join point (called knot), together with potential confounders. Knots represented change points, where the slope of the risk surface changes to account for potential departures from linearity. The set of spline regression parameters described the shape of the risk surface. For each cancer subsite, the optimal number of knots, their location, the regression and spline coefficients were jointly estimated within the Bayesian approach.^43^ Vague prior distributions were assumed on the regression and spline coefficients, with spike-and-slab priors on the spline coefficients managing the choice of the optimal number of knots within a modified Stochastic Search Variable Selection approach.^44^ The Markov Chain Monte Carlo (MCMC)-type NUTS (No-U-Turn Sampler) algorithm^8,45,46^ allowed to implement the Stochastic Search Variable Selection approach for identifying the optimal number of knots and then to derive the final joint posterior distribution of all the parameters, with the optimal combination of number of knots previously identified. Convergence was tested by algorithm-specific and generic MCMC diagnostics, reporting low number of divergences, a R-hat statistic <1.05 for each parameter, and a generally high effective sample size, suggesting the chains efficiently explored the posterior distribution. For each subsite, the ORs and their 95% credible intervals (CIs) were derived from the corresponding (final) posterior distribution. The ORs were presented through three-dimensional plots that displayed the surface of risk for any combination of alcohol drinking intensity and duration. In addition, we presented two-dimensional plots that displayed patterns of risks corresponding to one variable exposure for fixed levels of the other exposure. All the models were fitted with the full set of potential confounders, i.e. sex, age, study, race, education, cigarette-smoking status, cigarette-smoking intensity, cigarette-smoking duration and pipe and cigar status (Supplementary Table S2); “Never drinkers” were assumed as the reference category. Calculations were carried out using the open-source Stan program^47^ within the open-source R program.^48^

## Results

Study subjects were predominantly males (70.7%); the median age was 58 years for controls and for all cases together. Current smoking was reported in the majority of cancer patients (51.5% of oral cavity, 52.4% of oropharyngeal cancers, 63% of hypopharyngeal and 61.2% of laryngeal cancers), but not in the controls (24.2%; Supplementary Table S2).

In the study population (Table 1), patients with cancer of the oral cavity who were current drinkers drank at higher intensities (but not for a longer time period) than controls. The proportion of never drinkers was much lower among patients with oropharyngeal (17.4%), hypopharyngeal (8.9%) and laryngeal (17.8%) cancers; drinking habits in these cancers showed a higher intensity and a longer duration.

**Table 1:** Distribution of cases of oral cavity, oropharyngeal, hypopharyngeal and laryngeal cancers, and controls according to intensity and duration of alcohol drinking in current drinkers.

	Controls		Oral cavity		Oropharynx		Hypopharynx		Larynx	
	*n*	(%)	*n*	(%)	*n*	(%)	*n*	(%)	*n*	(%)
Total	24,234		4085		3359		983		3340	
Never drinkers	7873	(32.5)	1353	(33.1)	583	(17.4)	87	(8.9)	593	(17.8)
Drinking intensity (drinks/day)										
≤1	6921	(28.6)	757	(18.5)	801	(23.8)	105	(10.7)	583	(17.5)
>1–3	5470	(22.6)	805	(19.7)	787	(23.4)	242	(24.6)	771	(23.1)
>3–10	3970	(16.4)	1170	(28.6)	1188	(35.4)	549	(55.8)	1393	(41.7)
Drinking duration (years)										
1–30	6218	(25.7)	975	(23.9)	889	(26.5)	217	(22.1)	647	(19.4)
31–40	5061	(20.9)	920	(22.5)	1049	(31.2)	337	(34.3)	986	(29.5)
41–54	5082	(21.0)	837	(20.5)	838	(24.9)	342	(34.8)	1114	(33.4)
Age at start drinking (years)										
≤18	5482	(22.6)	1047	(25.6)	1172	(34.9)	324	(33.0)	1050	(31.4)
19–25	7016	(29.0)	1110	(27.2)	1126	(33.5)	423	(43.0)	1209	(36.2)
26–35	2435	(10.0)	332	(8.1)	328	(9.8)	106	(10.8)	331	(9.9)
>35	1428	(5.9)	243	(5.9)	150	(4.5)	43	(4.4)	157	(4.7)

**Fig. 1:**
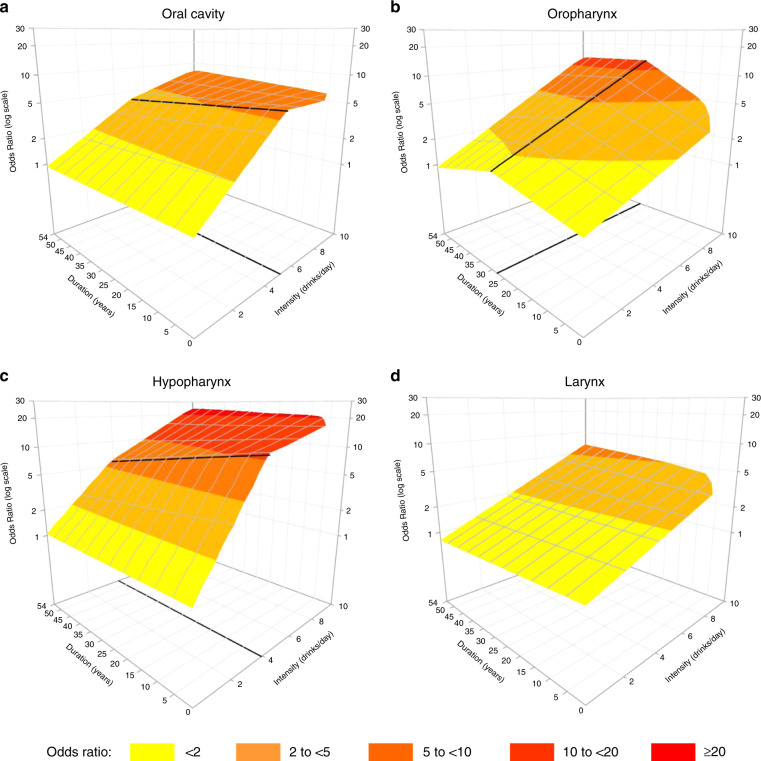
Cancer risk for the joint exposure to drinking intensity and duration (3D representation). Bivariate spline model’s estimates of odds ratios of oral cavity (**a**), oropharyngeal (**b**), hypopharyngeal (**c**), and laryngeal (**d**) cancers in current drinkers for the joint effect of intensity and duration of alcohol consumption. On the grid, black thicker lines represent knot locations, at 5 drinks/day for oral cavity, at 4 drinks/day for hypopharyngeal cancer and at 28 years for oropharyngeal cancer.

The surfaces of HNC risk for the joint exposure to drinking intensity and duration were displayed in Fig. 1. For all subsites, the risk steeply increased with increasing number of drinks/day, with no appreciable threshold effect at lower intensities. The risk peaked at the higher levels of duration and intensity (i.e. for people drinking 10 drinks/day for 54 years) for all subsites, reaching ORs of 8.0 (95% CI: 4.6–13) for oral cavity, 12.9 (95% CI: 7.2–23.7) for oropharynx, 25.0 (95% CI: 11.6–51.5) for hypopharynx and 6.6 (95% CI: 4.9–9) for larynx. For oral cavity (Fig. 1a) and hypopharynx (Fig. 1c), the risk flattened after 5 and 4 drinks/day, respectively. Moreover, the risk surfaces for cancers of the oral cavity, hypopharynx and larynx (Fig. 1a, c, and d) suggested no effect of drinking duration in addition to intensity: the risk remained stable when duration increased, for fixed levels of intensity. For oropharyngeal cancer (Fig. 1b), the risk increased with increasing years of duration up to 28 years, flattening thereafter; this effect was more marked at higher intensities. A sensitivity analysis conducted excluding only extremely high values (i.e. intensity >28 drink/day or duration >61 years, 1% of study subjects) showed similar results. The same analyses were further conducted in strata of gender (Supplementary Fig. S2): risk surfaces were similar in shape to those in the main analysis, even if cancer risk was slightly higher for women than for men. The subgroup analysis was not performed for the hypopharynx subsite, due to low number of cases.

**Fig. 2:**
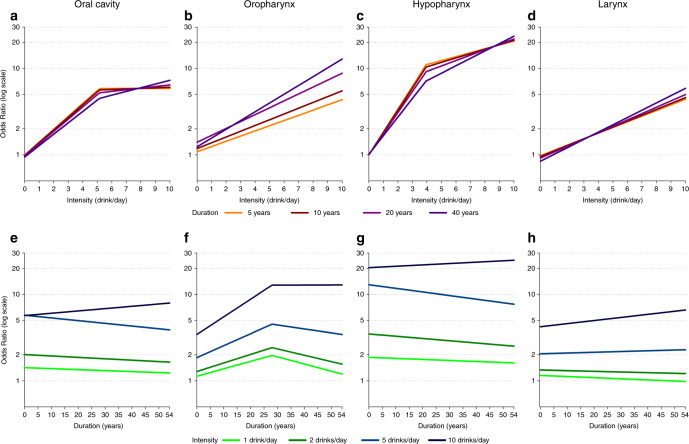
Cancer risk for the joint exposure to drinking intensity and duration (2D representation). Bivariate spline model’s estimates of odds ratios of oral cavity, oropharyngeal, hypopharyngeal and laryngeal cancers in current drinkers for alcohol intensity and fixed levels of alcohol duration (**a**–**d**) and for duration and fixed levels of intensity (**e**–**h**).

The same effects between alcohol intensity and duration across HNC subsites are also shown in Fig. 2, which presents the risk for increasing intensities at defined duration levels (upper panels), and the risk for increasing durations at defined levels of intensity (lower panels). For cancers of the oral cavity, hypopharynx, and larynx (Fig. 2a, c, and d), the curves for intensity at different durations were largely overlapping and showed an upward trend. This indicated that duration did not substantially modify cancer risk, which was mainly driven by drinking intensity. Figure 2e, g, and h confirmed this conclusion, showing generally flat curves for the three levels of intensities up to 5 drinks/day, as also suggested by the CIs (Supplementary Table S3); a modest upward trend was present at the highest intensity level (i.e. 10 drinks/day). Differently, a joint effect of intensity and duration was found for oropharyngeal cancer risk: the risk increased with increasing intensities, but higher levels of duration raised up the curves to the highest risk (Fig. 2b); duration increased oropharyngeal cancer risk up to ~28 years (Fig. 2f), although the contribution of duration to the risk was particularly evident at the highest level of intensity (i.e. 10 drinks/day).

## Discussion

The present analyses show that, consistently between genders, drinking intensity was the predominant measure of alcohol affecting the risk of oral cavity, hypopharyngeal and laryngeal cancers, whereas the contribution of duration, for fixed alcohol intensities, was modest. Notably, this suggests that drinking alcohol beverages, even for a short period, increases the risk at these cancer subsites and that duration of alcohol use has little or no consistent effect on the risk of these cancers. Differently, there was a joint effect of drinking intensity and duration in determining oropharyngeal cancer risk.

The direct association between alcohol intensity and HNC risk has been extensively described^2,6,49^ and potential mechanisms have been proposed.^5,50^ Ethanol is oxidised to alcohol acetaldehyde (AA), which is a recognised carcinogen.^2^ Alcohol may also have a local effect, acting as a solvent of cell membranes to enhance the penetration of carcinogens, notably those from tobacco smoking, into the mucosa.^50^ Further, nutritional deficiencies may occur in alcoholics.^50^

The relationship between drinking duration and HNC risk was more complex, with a clear association with oropharyngeal cancer risk up to ~28 years of drinking. These results are in agreement with previous findings derived from a standard approach on a smaller set of INHANCE studies (15 studies) including never smokers only, which showed no association with alcohol duration in all HNC subsites but hypo/oropharynx.^14^ Furthermore, the application of a different statistical approach^7^ on the 15 INHANCE studies supported the presence of a stronger association with intensity than with duration for HNC risk. Although the lack of association with duration may seem counterintuitive, it has been reported in oesophageal adenocarcinomas, another alcohol-related cancer, in a large pooled analysis on 12 case-control studies.^51^ Although these results did not allow to draw biological interpretations, they suggest that alcohol intake acts as a late-stage carcinogen.^52^

A major limitation of the present study was information bias, which may have occurred as a consequence of the complexity of lifetime drinking patterns. Changes in the intensity, in type of alcohol beverages, and temporary quitting are more frequent for alcohol drinking than for other lifestyle habits,^53^ such as tobacco smoking; lifetime patterns may have an impact on the risk of cancer.^54^ Therefore, misclassification may have occurred for both intensity and duration. The calculation of lifetime average alcohol intake may have protected against this source of bias, thus not allowing the investigation of specific drinking patterns (e.g. infrequent heavy binge drinking). In addition, the use of linear bidimensional spline models may have contributed too, as they are quite robust with respect to small variations in the predictors, as compared to bidimensional splines of higher degrees. To test for model robustness, we adopted different solutions of truncation or approximation of drinking intensity and duration, and the resulting surface estimates were similar. Further, self-reporting of drinking habits may have led to additional information bias, since higher values of intensity and duration are more prone to inaccurate reporting.^55,56^ To reduce information bias and residual confounding at the extreme values of the exposure distributions, we excluded subjects reporting higher (>95th percentiles) drinking intensity and/or duration from the present analysis; however, this could have led to a reduced study power and differential exclusion of cases and controls. Finally, our Bayesian approach was computationally time consuming, requiring dedicated server devices.

Although risk estimates were adjusted for tobacco smoking (considering cigarettes, cigars and pipes), some residual confounding may remain. An analysis among never smokers would rule out possible residual confounding due to tobacco smoking. However, considering that the present logistic models includes several covariates, they require large sample sizes to produce precise estimates; thus, we were unable to conduct this subgroup analysis with sufficient precision. Nonetheless, the previously cited INHANCE analysis on never smokers^14^ reported results similar to the current ones, with HNC risk generally increasing with alcohol intensity and no dose-response relation with drinking duration. Further, the lack of information on infection with human papilloma virus (HPV) has to be accounted among study limitations, considering the recognised role of HPV in oropharyngeal cancer.^57^ Unfortunately, HPV status was not collected in the majority of studies, since they were conducted before the awareness of the HPV role in oropharyngeal cancer. International representativeness is guaranteed by the large dataset including studies from different geographical areas. On the other hand, the inclusion of heterogeneous populations, in particular that of different genetic origin, may have led to estimation bias. Compared to other populations, East Asians have a much higher frequency of A allele of ALDH2 rs671,^58^ which slows acetaldehyde metabolism, thus increasing alcohol-related risk. However, the exclusion of East Asian studies^16,33^ did not substantially modify the risk estimates.

Results of the present study are strengthened by the availability of information on several potential confounding factors. In addition, we applied a Bayesian approach to jointly estimate the optimal knot locations and the ORs of HNC for the joint effect of our continuous predictors.^8^ As compared to the companion paper on cigarette-smoking intensity and duration, in the current application the optimal number of knots was estimated within a two-step procedure including the Stochastic Search Variable Selection approach.^43^ To our knowledge, this is the first time that a similar approach is applied within the context of spline models in epidemiology.

In conclusion, findings of the present study indicate that the risk of cancer of the oral cavity, hypopharynx and larynx increases with drinking intensity, whereas the role of duration is complex. The trend is linear for larynx, but it showed a plateau at the highest intensity for cancer of the oral cavity and hypopharynx. The joint effect of intensity and duration increases the risk of oropharyngeal cancer. In addition, no threshold effect is evident at the lowest doses. Although abstinence from alcohol drinking would be the ultimate goal to reduce HNC incidence, these findings suggest that any reduction in alcohol intake^59^ would be an effective strategy to mitigate HNC risk, as well as the risk of few other neoplasms.^60^

## Supplementary information

Supplementary Tables S1-S2

## Data Availability

Data are available for scientific purposes upon reasonable request to the corresponding authors.
