# Supplementary material for: Alcohol drinking and head and neck cancer risk: the joint effect of intensity and duration
Source: Br J Cancer. 2020 Aug 24;123(9):1456–63. doi: 10.1038/s41416-020-01031-z (PMC7592048; doi:10.1038/s41416-020-01031-z)
Supplement: Supplementary file 1 — Supplementary Tables S1-S2 [file 41416_2020_1031_MOESM1_ESM.pdf]

Supplementary Table S1. Characteristics of the individual studies from the International Head and Neck Cancer Epidemiology (INHANCE) Consortium used in the current analysis. INHANCE consortium.

Study name, location	Case source	Age eligibility	Case participation rate, %	Control source	Control participation rate, %	Matched factors	Recruitment period
**Milan (1984-1989), Italy**	Hospital	<80	95[a]	Hospital - unhealthy	95[a]	--	1984-1989
**Aviano, Italy**	Hospital	>18	>95[a]	Hospital - unhealthy	95[a]	--	1987-1992
**France (1987-1992), France**	Hospital	NA	95[a]	Hospital - unhealthy	95[a]	Age, sex, hospital	1987-1992
**Italy Multicenter**	Hospital	18-80	>95	Hospital - unhealthy	>95	--	1990-1999
**Switzerland**	Hospital	<80	>95	Hospital - unhealthy	>95	--	1991-1997
**New York, NY, USA (multicenter)**	Hospital	21-80	91	Hospital- unhealthy	97	Age, sex, hospital, year of interview	1981-1990
**Seattle (1985-1995), WA, USA**	Cancer registry	18-65	54.4,63.3[b]	Random digit dialing	63.0,60.9[b]	Age, sex	1985-1995
**Iowa, IA, USA**	Hospital	>18	87	Hospital - unhealthy	92	Age, sex	1993-2006
**North Carolina (1994-1997), NC, USA**	Hospital	>17	88	Hospital - unhealthy	86	Age, sex	1996-1997
**Tampa, FL, USA**	Hospital	≥18	98	Hospital - noncancer	90	Age, sex, ethnicity	1994-2003
**Los Angeles, CA, USA**	Cancer registry	18-65	49	Neighborhood	68	Age, sex, neighborhood	1999-2004
**Houston, TX, USA**	Hospital	≥18	95	Hospital visitors	>80	Age, sex, ethnicity	2001-2006
**Puerto Rico**	Cancer registry	21-79	71	Residential records (healthy population)	83	Age, sex	1992-1995
**Latin America**	Hospital	15-79	95	Hospital - unhealthy	86	Age, sex, ethnicity, city	2000-2003
**International Multicenter, IARC**	Hospital	NA	88,7	Hospital/Community	87,3	Age, sex, center	1992-1997
**Boston, MA, USA**	Hospital	≥18	88,7	Residential records	48,7	Age, sex, neighborhood	1999-2003
**Sao Paulo, Brazil**	Hospital	NA		Hospital-unhealthy		age, sex, city of residence, hospital	2002-2007
**New York (MSKCC), NY, USA**	Hospital	NA	--	Blood donors	--	Age, sex	1992-1994
**Seattle-Leo, WA, USA**	Cancer registry	20-74	81	Random digit dialing	75	Age, sex	1983-1987
**Western Europe (ARCAGE)**	Hospital	NA	82	Hospital-unhealthy (population-based for UK centers)	68	Age, sex, ethnicity, city	2000-2005

**Japan (2001-2005)**	Cancer Hospital	20-79	97,00	Hospital - unhealthy	97,00	Age, sex	2001-2005
**North Carolina (2002-2006), NC, USA**	Cancer registry	20-80	82	DMV files	61	Age, sex, ethnicity	2002-2006
**France Multicenter (2001-2007)**	Cancer registry	<=75	82,5	Random digit dialing	80,6	Age, sex, region	2001-2007
**Baltimore, MD, USA**	Hospital	NA	100	Hospital - benign conditions	70	age, sex, HPV status	2000-2005
**Beijing, China**	Hospital	18-80	100	Hospital	100	Age, sex	1988-1989
**Milan (2006-2009), Italy**	Hospital	18-80	>95	Hospital	>95	----	2006-2009

ABBREVIATIONS: ARCAGE: Alcohol-Related Cancers And Genetic susceptibility in Europe; DMV: Department of Motor Vehicles; HPV: Human Papilloma Virus; IARC: International Agency for Research on Cancer; MSKCC: Memorial Sloan Kettering Cancer Center; NA: Not Available.

[a] Participation rate was not formally assessed, estimated response rate reported.
[b] Two response rates are reported because data were collected in two population-based case-control studies, the first from 1985 to 1989 among men and the second from 1990 to 1995 among men and women.

Supplementary Table S2. Distribution of cases of oral cavity, oropharyngeal, hypopharyngeal, and laryngeal cancers, and controls according to selected variables. INHANCE Consortium

	Controls		Oral cavity		Oropharynx		Hypopharynx		Larynx	
	**n**	**(%)**	**n**	**(%)**	**n**	**(%)**	**n**	**(%)**	**n**	**(%)**
**TOTAL**	24,234	(100)	4085	(100)	3359	(100)	983	(100)	3340	(100)
**Sex**[a]										
Female	7533	(31.1)	1552	(38.0)	780	(23.2)	138	(14.0)	533	(16.0)
Male	16,701	(68.9)	2533	(62.0)	2579	(76.8)	845	(86.0)	2807	(84.0)
**Age (years)**[a]										
<40	1720	(7.1)	284	(7.0)	124	(3.7)	7	(0.7)	68	(2.0)
40 to 44	1711	(7.1)	234	(5.7)	211	(6.3)	38	(3.9)	116	(3.5)
45 to 49	2479	(10.2)	442	(10.8)	449	(13.4)	104	(10.6)	294	(8.8)
50 to 54	3547	(14.6)	645	(15.8)	617	(18.4)	184	(18.7)	493	(14.8)
55 to 59	4217	(17.4)	743	(18.2)	727	(21.6)	211	(21.5)	682	(20.4)
60 to 64	3944	(16.3)	670	(16.4)	542	(16.1)	182	(18.5)	671	(20.1)
65 to 69	3431	(14.2)	492	(12.0)	409	(12.2)	144	(14.6)	603	(18.1)
70 to 74	2304	(9.5)	336	(8.2)	206	(6.1)	92	(9.4)	310	(9.3)
≥75	881	(3.6)	239	(5.9)	74	(2.2)	21	(2.1)	103	(3.1)
**Study name**										
Aviano	652	(2.7)	52	(1.3)	71	(2.1)	38	(3.9)	91	(2.7)
Baltimore	162	(0.7)	36	(0.9)	83	(2.5)	4	(0.4)	31	(0.9)
Beijing	380	(1.6)	377	(9.2)						
Boston	400	(1.7)	90	(2.2)	145	(4.3)	22	(2.2)	68	(2.0)
France (1987-1992)	194	(0.8)	41	(1.0)	50	(1.5)	27	(2.7)	127	(3.8)
France Multicenter (2001-2007)	3149	(13.0)	343	(8.4)	489	(14.6)	275	(28.0)	368	(11.0)
Houston	712	(2.9)	182	(4.5)	306	(9.1)	30	(3.1)	108	(3.2)
International Multicenter	1297	(5.4)	640	(15.7)	224	(6.7)			2	(0.1)
Iowa	550	(2.3)	173	(4.2)	97	(2.9)	7	(0.7)	60	(1.8)
Italy Multicenter	2246	(9.3)	126	(3.1)	193	(5.7)	77	(7.8)	324	(9.7)
Japan (2001-2005)	2796	(11.5)	100	(2.4)	63	(1.9)	63	(6.4)	80	(2.4)
Latin America	993	(4.1)	199	(4.9)	154	(4.6)	66	(6.7)	367	(11.0)
Los Angeles	815	(3.4)	34	(0.8)	93	(2.8)	8	(0.8)	51	(1.5)
Milan (1984-1989)	1450	(6.0)	40	(1.0)	32	(1.0)	25	(2.5)	229	(6.9)
Milan (2006-2009)	568	(2.3)	64	(1.6)	10	(0.3)	9	(0.9)	145	(4.3)
MSKCC	89	(0.4)	34	(0.8)	5	(0.1)	5	(0.5)	24	(0.7)
New York Multicenter	1098	(4.5)	303	(7.4)	286	(8.5)	17	(1.7)	123	(3.7)
North Carolina (1994-1997)	157	(0.6)	28	(0.7)	24	(0.7)	10	(1.0)	25	(0.7)
North Carolina (2002-2006)	962	(4.0)	99	(2.4)	218	(6.5)	33	(3.4)	239	(7.2)
Puerto Rico	278	(1.1)	25	(0.6)	35	(1.0)	10	(1.0)		
Sao Paulo	1145	(4.7)	347	(8.5)	111	(3.3)	52	(3.4)	224	(6.7)
Seattle (1985-1995)	502	(2.1)	160	(3.9)	113	(3.4)				
Seattle-Leo	431	(1.8)	113	(2.8)	85	(2.5)	29	(3.0)	116	(3.5)
Switzerland	823	(3.4)	109	(2.7)	110	(3.3)	72	(7.3)	111	(3.3)
Tampa	738	(3.0)	16	(0.4)	37	(1.1)	1	(0.1)	38	(1.1)
Western Europe	1647	(6.8)	354	(8.7)	325	(9.7)	103	(10.5)	389	(11.6)

Continues

Supplementary Table S2. Continued

**Race**[a]										
Asian and Pacific Islanders	3779	(15.6)	833	(20.4)	132	(3.9)	64	(6.5)	87	(2.6)
Black	631	(2.6)	149	(3.6)	108	(3.2)	30	(3.1)	109	(3.3)
Hispanic	304	(1.3)	29	(0.7)	37	(1.1)	2	(0.2)	7	(1.0)
Others and Brazilians	2267	(9.3)	564	(13.8)	301	(9.0)	121	(12.3)	598	(17.9)
White	17,253	(71.2)	2510	(61.4)	2781	(82.8)	766	(77.9)	2514	(75.3)
**Education**[a]										
No education	980	(4.0)	472	(11.6)	55	(1.6)	32	(3.3)	94	(2.8)
≤Junior high school	8408	(34.7)	1341	(32.8)	969	(28.8)	409	(41.6)	1495	(44.8)
Some high school	4340	(17.9)	787	(19.3)	715	(21.3)	257	(26.1)	632	(18.9)
High school graduate	3097	(12.8)	567	(13.9)	510	(15.2)	121	(12.3)	458	(13.7)
Technical sch., some college	3680	(15.2)	513	(12.6)	559	(16.6)	90	(9.2)	408	(12.2)
≥College graduate	3729	(15.4)	405	(9.9)	551	(16.4)	74	(7.5)	253	(7.6)
**Cigarette smoking status**										
Never user	10,570	(43.6)	1263	(30.9)	660	(19.6)	72	(7.3)	223	(6.7)
Former user	7804	(32.2)	717	(17.6)	939	(28.0)	292	(29.7)	1072	(32.1)
Current user	5860	(24.2)	2105	(51.5)	1760	(52.4)	619	(63.0)	2045	(61.2)
**Cigarette smoking intensity (cigarettes/day)**										
≥1-10	4046	(16.7)	475	(11.6)	452	(13.5)	138	(14.0)	352	(10.5)
>10-20	5837	(24.1)	1254	(30.7)	1157	(34.4)	391	(39.8)	1366	(40.9)
>20-30	1826	(7.5)	567	(13.9)	503	(15.0)	186	(18.9)	657	(19.7)
>30-40	1301	(5.4)	373	(9.1)	412	(12.3)	149	(15.2)	503	(15.1)
>40	654	(2.7)	153	(3.7)	175	(5.2)	47	(4.8)	239	(7.2)
**Cigarette smoking duration (years)**										
1-10	1801	(7.4)	132	(3.2)	166	(4.9)	20	(2.0)	72	(2.2)
11-20	2696	(11.1)	228	(5.6)	220	(6.5)	56	(5.7)	169	(5.1)
21-30	3426	(14.1)	572	(14.0)	562	(16.7)	172	(17.5)	523	(15.7)
31-40	3327	(13.7)	1004	(24.6)	968	(28.8)	347	(35.3)	1084	(32.5)
>40	2414	(10.0)	886	(21.7)	783	(23.3)	316	(32.1)	1269	(38.0)
**Cigar smoking status**										
Never user	19,438	(80.2)	3,347	(81.9)	2991	(89.0)	853	(86.8)	3023	(90.5)
Current user	1004	(4.1)	186	(4.6)	248	(7.4)	57	(5.8)	197	(5.9)
Unknown	3792	(15.6)	552	(13.5)	120	(3.6)	73	(7.4)	120	(3.6)
**Pipe smoking status**										
Never user	20,269	(83.6)	3719	(91.0)	3073	(91.5)	862	(87.7)	3074	(92.0)
Current user	1125	(4.6)	242	(5.9)	205	(6.1)	52	(5.3)	164	(4.9)
Unknown	2840	(11.7)	124	(3.0)	81	(2.4)	69	(7.0)	102	(3.1)

ABBREVIATIONS: MSKCC: Memorial Sloan Kettering Cancer Center.

[a] We excluded subjects with missing information on age, sex, race and cigarette smoking habits. Missing values for education were imputed according to the INHANCE protocol.

Supplementary Table S3. Odds Ratio (OR) and 95% Credible Intervals (CI) of cases of oral cavity, oropharyngeal, hypopharyngeal, and laryngeal cancers, for selected levels of alcohol intensity and duration. INHANCE Consortium

		Oral cavity		Oropharynx		Hypopharynx		Larynx	
		**OR**	**95% CI**	**OR**	**95% CI**	**OR**	**95% CI**	**OR**	**95% CI**
**Intensity (drinks/day)**	**Duration (Years)**								
**1**	**5**	1.40	1.30-1.51	1.25	1.14-1.36	1.84	1.58-2.20	1.14	1.08-1.20
	**10**	1.38	1.29-1.48	1.38	1.26-1.52	1.81	1.55-2.17	1.12	1.06-1.19
	**20**	1.35	1.25-1.45	1.67	1.46-1.99	1.76	1.48-2.13	1.09	1.01-1.17
	**40**	1.28	1.14-1.44	1.56	1.16-2.73	1.67	1.28-2.21	1.03	0.90-1.16
**2**	**5**	1.97	1.72-2.29	1.44	1.19-1.70	3.35	2.52-4.83	1.32	1.20-1.46
	**10**	1.94	1.71-2.21	1.61	1.39-1.86	3.25	2.49-4.55	1.31	1.20-1.44
	**20**	1.86	1.68-2.08	2.01	1.74-2.40	3.07	2.42-4.14	1.29	1.18-1.41
	**40**	1.73	1.54-1.94	1.97	1.45-2.73	2.73	2.10-3.64	1.25	1.10-1.40
**5**	**5**	5.52	3.92-7.97	2.19	1.35-3.32	11.95	6.94-25.47	2.07	1.63-2.65
	**10**	5.32	3.96-7.32	2.56	1.81-3.53	11.41	6.93-23.31	2.09	1.70-2.60
	**20**	4.95	3.95-6.27	3.49	2.88-4.38	10.38	6.85-19.58	2.14	1.83-2.52
	**40**	4.29	3.59-5.16	3.93	2.92-6.03	8.58	6.06-15.20	2.23	1.96-2.52
**10**	**5**	6.07	1.99-13.49	4.43	1.64-10.13	21.62	4.45-82.86	4.37	2.71-7.15
	**10**	6.23	2.32-12.67	5.55	2.74-10.53	21.65	5.31-75.35	4.56	3.01-6.98
	**20**	6.54	3.19-11.13	8.79	6.16-12.65	21.91	7.43-65.55	4.95	3.66-6.77
	**40**	7.32	4.75-10.31	12.48	8.89-22.72	23.29	11.85-51.70	5.88	4.83-7.16

Legends to supplementary figures

Supplementary Figure S1. Flow-chart of subjects' selection process. INHANCE Consortium.

Supplementary Figure S2. Bivariate spline models estimates of odds ratios of oral cavity, oropharyngeal, and laryngeal cancers in current drinkers for the joint effect of intensity and duration of alcohol consumption, in strata of gender. On the grid, black thicker lines represent knot locations, at 5 drinks/day for oral cavity in men, and at 30 years for oropharyngeal cancer in men. INHANCE Consortium.

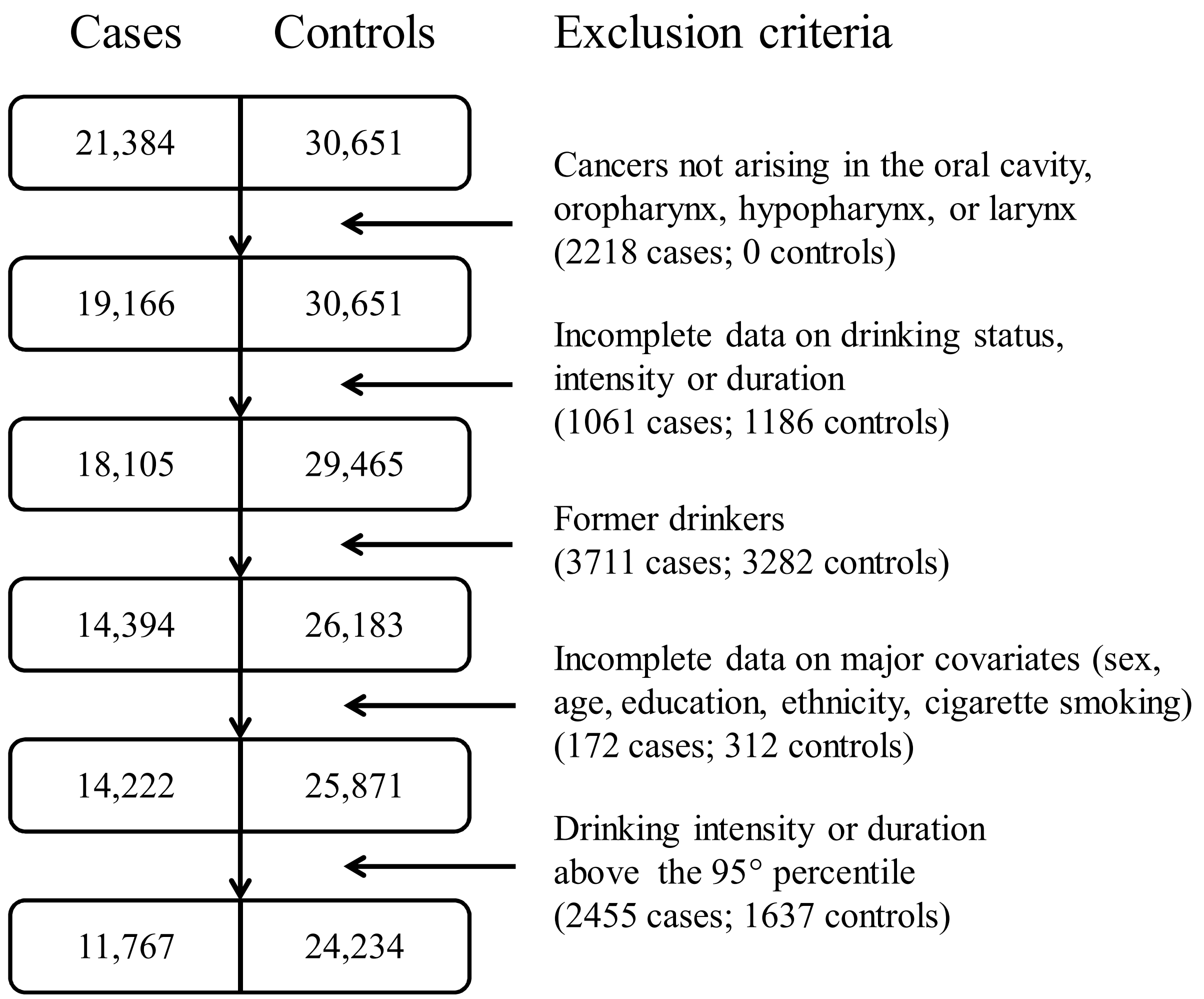
Cases
Controls
Exclusion criteria
21,384
30,651
Cancers not arising in the oral cavity, oropharynx, hypopharynx, or larynx (2218 cases; 0 controls)
19,166
30,651
Incomplete data on drinking status, intensity or duration (1061 cases; 1186 controls)
18,105
29,465
Former drinkers (3711 cases; 3282 controls)
14,394
26,183
Incomplete data on major covariates (sex, age, education, ethnicity, cigarette smoking) (172 cases; 312 controls)
14,222
25,871
Drinking intensity or duration above the 95° percentile (2455 cases; 1637 controls)
11,767
24,234

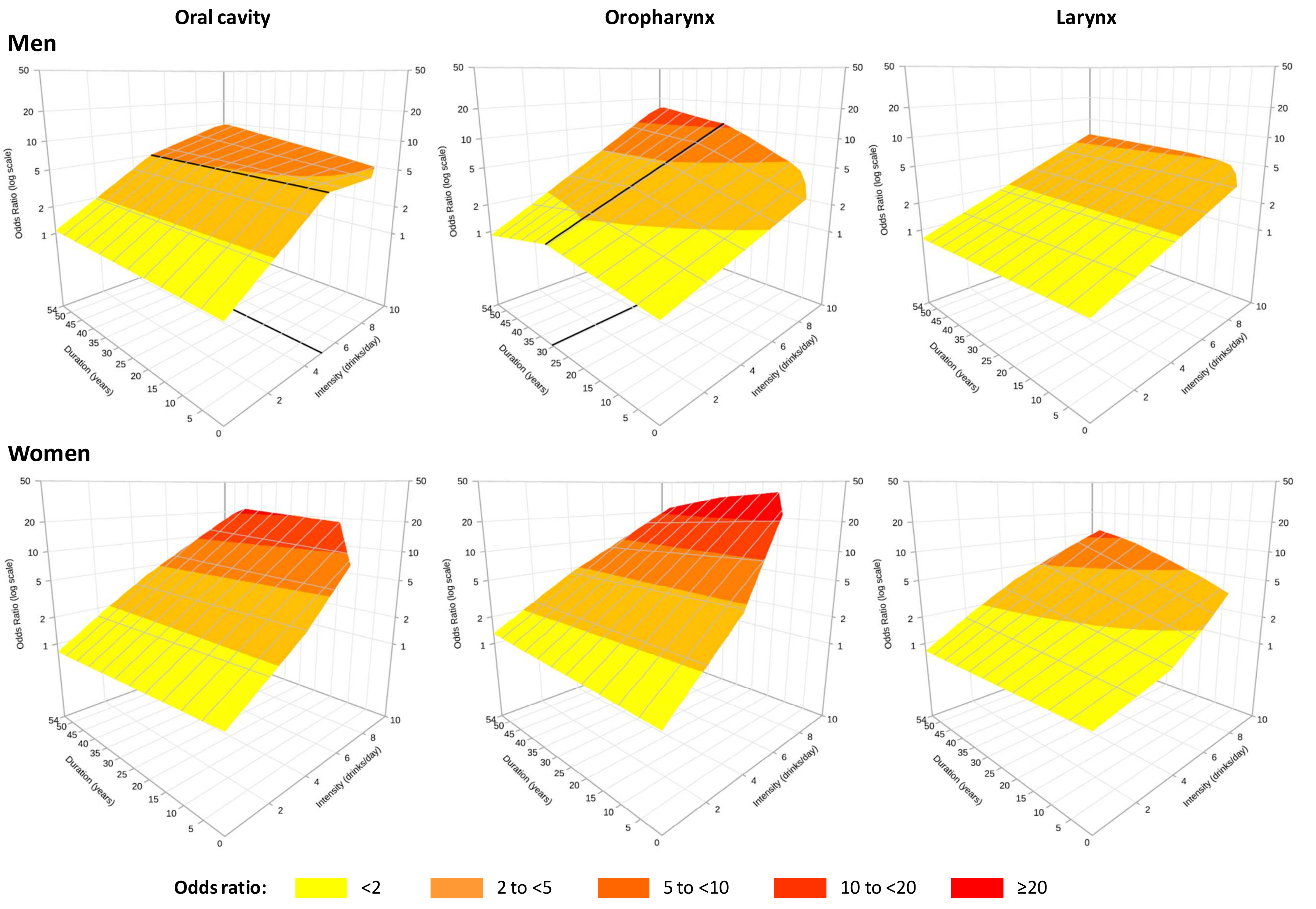

Oral cavity
Oropharynx
Larynx
Men
Women
Odds Ratio (log scale)
Duration (years)
Intensity (drinks/day)
Odds ratio:
<2
2 to <5
5 to <10
10 to <20
≥20
